# Book Reviews

**Published:** 1803-08-01

**Authors:** 


### CRITICAL ANALYSIS

OF THE

### RECENT PUBLICATIONS

ON THE

### DIFFERENT BRANCHES OF PHYSIC, SURGERY, AND MEDICAL PHILOSOPHY.

*The Edinburgh Practice of Physic, Surgery, and Midwifery, preceded by an Abstract of the Theory of Medicine and the Nosology of* Dr. CULLEN, *and including upwards of Six Hundred authentic Formulæ, &c.* with twenty quarto plates. A new edition in five volumes, viz. the two first on Medicine, the third and fourth on Surgery, and the fifth on Midwifery. 1803.

THE numerous additions with which the present edition of this valuable compilation has been enriched, and the quantity of medical knowledge which it contains, will justify us in bestowing on it more notice than a mere compilation might at first seem to demand. The title by which this work has long been known, is, "The Edinburgh Practice," by which is meant, a summary of all the knowledge, theoretical as well as practical, taught and practised at the Edinburgh School of Physic. A work of this kind, if executed with fidelity, cannot fail of being extensively useful, provided too much be be not expected from it. The perusal, or even study of a work of this kind, can no more make an accomplished physician than the study of Blackstone's Commentaries can make a profound lawyer; but there is a large class of medical students and practitioners to whom this Compendium must be desirable, as to them it offers the only probable method of coming at much valuable information, which it is better to know imperfectly, than not to know at all.

In the preface, the Editor speaks of the plan and performance of his task with modesty and propriety. The parts which are the most considerably increased in the present edition, are *the Cases and the Formulæ*, both of which add most materially to its utility as a practical guide. It was hardly necessary, we think, to introduce any justification of extending the plan to Surgery and Midwifery, as well as Medicine; the education of a physician includes all these branches, and, what is more to the point, the readers to whom this work will be particularly acceptable, are, we conceive, the largest and the truly respectable body of practitioners in this kingdom, those who unite in practice all the branches of the Hippocratic art.

A sketch of the history of Medicine forms the Introduction. It is brought down to the time of Harvey, generally; and a very good view is given of the leading points which distinguished the practice of Hippocrates. From the time of Harvey, this history only flows in one channel, namely, the rise and progress of the Edinburgh School of Physic. We do not mean, however, to insinuate any accusation of improper partiality in thus confining the subject; the writer gives fairly and candidly that which he professes to give, a History of this celebrated Medical School; and perhaps, in the compass which he allows himself, a more extensive view of the modern theories of Physic, (though always equal to and generally far surpassing in excellence the crude conceptions of the antients) would only perplex by excess of brevity.

The first chapter contains a view of the Theory of Medicine, an account of the principal functions of the body, and of some of the most popular systems. It will be sufficient for our readers to say, that the account of the functions of the body is given from an elementary and elegant work in the Edinburgh School, Dr. Gregory's Conspectus Medicinæ Theoreticæ, and that the Systems which are explained are those of Cullen and Brown. The latter is introduced with no great respect; but it is not now that the Brunonian system will be thrown back by such disparaging expressions as, "many of our readers will little expect to see Dr. Brown's opinions seriously noticed,"—"dignified by its admirers with the title of a system,"—"converts to the doctrine are totally unable to recollect when or how they were converted;" and others of the same stamp. Brunonianism was indeed first adopted by young students, and it was long before it had acquired that rank among medical systems to which its ingenuity and the abilities of its inventor justly entitled it; but the *young* men who enrolled themselves among the first supporters of Brown's opinions, were some of the highest ornaments to the Edinburgh School, and the system itself has gradually wrought a change of great extent both in the general practice of physic, and even in the language of medical reasoning.

As some system of Nosology must be taken as the text-book for descriptions of diseases, the Editor (as will be readily supposed) follows that of Dr. Cullen, a translation of which is prefixed, for the benefit of those readers (perhaps not a few) to whom the perusal of the original would be attended with some trouble.

Therapeutics, or the Practice of Medicine next succeed, and occupy the remainder of this and the succeeding volume. Febrile diseases are the first given; and on this subject, of infinite importance, great attention is bestowed. Dr. Darwin's system of Fever, taken from the Zoonomia, is first however introduced, as an appendix to the chapter on the theory of Fever, where indeed it would have come in with much more propriety along with the Brunonian system, which it so closely resembles. Though the Editor takes Cullen for his guide in regulating the nosology, and makes ample use of the "First Lines," much more than the concise and often meagre descriptions of this author is required by the reader who would have recourse to such a work as the present as a guide to his practice; the editor therefore very properly has supplied the deficiencies of description by the valuable matter contained in the works of Lind, Fordyce, Huxham, Saunders, Currie, and many other eminent practitioners. Still, however, we are compelled to remark, that though a great mass of excellent matter is collected, it is merely thrown down upon the pages, without much method or system; long quotations are given from these writers, perfectly apposite indeed, but not interwoven into one clear and intelligible whole, nor arranged in that lucid order which is so necessary to assist the student, and to prevent that perplexity which must ensue from a multitude of counsellors. Full as the chapter on fever is, some omissions occur on points of practice, owing perhaps to the Editor confining himself too strictly within the limits of a mere compiler's duty. We might instance more particularly the use of certain remedies in typhus fever to fulfil peculiar indications, which we think might have been rendered much more conspicuous than in the pages before us. The particular use of opium in the spasmodic symptoms of the worst species of typhus; the advantage to be derived from a vigorous but cautious use of calomel, where much foulness of the intestinal canal is apprehended; the diaphoretic powers of ether, so much insisted on by Dr. Carmichael Smyth, and some other circumstances of the kind, would have added much to the intrinsic value of this part of the work, and might have beeen introduced in the place of some foreign matter of inferior importance. On turning over the method of purifying ships, apartments, and goods from the infection of fever, we had no doubt of finding due notice taken of the mineral acid vapours, and the superiority of this method over the old fumigations with wood, gunpowder, and aromatics.

aromatics. This we the more fully expected, as the excellent practice of cold affusion, so admirably inculcated and explained by Dr. Currie, is introduced with due attention. It was with no small surprize that we found not a single syllable on the acid vapours, though the fumigating processes recommended by Lind are detailed with great minuteness. The Editor has shewn himself too well acquainted with the actual state and modern history of physic to be ignorant of this improvement, why then has he not thought proper to mention it?

The two first volumes of this large and useful work are occupied by Medicine. We do not think it necessary to give any further analysis of their contents; all the principal diseases are noticed in general in a very ample manner, and the authorities consulted by the compiler are always respectable, and among those of established reputation, which are to be found in every library. This part of the subject does not admit of or require the illustration of plates. A single one is given, Mr. Watt's portable pneumatic apparatus for procuring the gasses for medicinal purposes. We shall decline our usual custom of giving a specimen of the execution of the work, as it can hardly be requisite in the present instance, and as the Editor has chosen that the readers should find not only the matter but the *very words* of Cullen, Lind, Fordyce, and many of the Correspondents that have long enriched our Journal with their Communications.

[ To be continued. ]

---

*A Second Treatise on the Bath Waters, comprehending their medicinal Powers in general, and particularly as they relate to the Cure of Dyspepsia, Gout, Rheumatism, Jaundice, and Liver Complaints, Chlorosis, cutaneous Eruptions, Palsy, &c.* By George Smith Gibbes, M.D. F.R.S. late Fellow of Magdalene College, Oxford. 8vo. pp. 120. Bath, 1803.

The first Treatise of this zealous advocate of Bath water, exhibited the analysis of this renowned spring with so much accuracy of chemical investigation, as to leave nothing further to be desired on this head as long as chemistry remains what it is. In the present work, Dr. Gibbes proceeds to its application, as appears from the title page, to all the principal diseases for which the Bath water is recommended. In so doing, something more is intended, and we may rather entitle this little work a defence of the virtues of Bath water against the attacks of Drs. Heberden, Saunders, and some other physicians, "who, residing at a distance from Bath, are not fully aware of its medicinal excellence." The controversy is certainly interesting, and the author defends his point with candour and urbanity. The sentiments of the late Dr. Heberden on the powers of Bath water, (an authority of no small weight, as far as authority ought to have weight on such subjects) strike directly at the root of the high reputation of this spring, by refusing to allow it more, or scarcely more, medicinal virtue than common water heated to the same temperature. In no part of the writings of this venerable physician is this opinion expressed so unequivocally as in his posthumous *Commentarii*, (article, *De aqua Bathonica)*. After lamenting the uncertainty of establishing *facts* in medicine, and giving in illustration the utterly contradictory opinions on the efficacy of Bath water, uttered by physicians of learning and sense, this is the inference which he deduces: "Profecto ut mihi videtur ubi medici prudentes in tam diversas sententias eunt, ibi censendum est hos fontes esse exiguarum vel nullarum virium." Nor is he more favourable to chemical analysis in general, "Taceo chemicorum analyses, ut parum idoneas ad vires quorumcumque medicamentorum explorandas."

Dr. Saunders, in the first edition of his valuable Treatise on the Liver, by extolling the power of simple warm water in circumstances of disease of this organ, in which Bath water is known to be serviceable, has contributed to throw some doubt on part of the supposed medicinal virtue of these springs. It is right, however, to observe, that this physician has, in the third edition of this work, retracted or modified this opinion in the following terms. "A more intimate acquaintance with the power of Bath water, and a minute inquiry into its sensible effects on the animal economy, having inclined me to think more favourably of its use, independently of its being merely an aqueous diluent." These, with the sentiments of some other medical observers on the subject, are the opinions which Dr. Gibbes aims to refute, chiefly by endeavouring to shew that these waters do really possess an actively sensible operation; that they are capable of producing as much positive mischief, when misapplied, as benefit when cautiously employed; and that the diseases where they are used, are of a nature to require powerful remedies, and, if we may so speak, to do credit to the healing art and the skill of the physician.

The chalybeate contained in this water must, after all, be the *only* foreign principle on which the medical chemist can rest with any security, and the author shews he is aware of this, by the stress which he lays on the peculiar mode of its combination with the water. He observes, "Reasoning a priori from the impregnation discoverable in the Bath waters by chemical agents, we certainly should not be led to suspect that they would have any great effect on the human frame. Experience, however, teaches us, that these waters possess very active powers when introduced into the body; and we are, I apprehend, authorized in attributing their effects to those substances in them, with which chemistry has made us acquainted. Because other chalybeate waters contain larger quantities of the metal, it is not to be supposed but that a different mode of impregnation may augment or vary the effects these produce on the animal economy. Who could suppose that a little addition of one of the principles which compose our atmosphere, could change calomel into corrosive sublimate, or that the imperceptible operation operation going on in the glands of vegetables, should produce from water, air, and earth, the deadliest poisons; poisons which, when once admitted into the animal system, cause instantaneous death? It is not, I contend, from the fewness of the principles, or from their quantity, that we can declare these waters to possess no activity, or, possessing it, to determine that it does not arise from the substances as yet discovered in them."

And again, "The chalybeate impregnation is of so delicate a kind, and so different from that of all other waters, as nearly to have eluded the nicest chemical research. The almost volatile state of the iron must give it an activity upon the human system, fully equal to what we see produced by these waters. Besides that our taste and smell discover in these waters, when fresh from the spring, a very perceptible chalybeate impregnation, chemical agents constantly detect in that state a larger proportion of iron in them. It is also found that the more delicate the reagent is, the greater is the quantity of iron discoverable. In my former treatise, I mentioned the reasons which induced me to believe that the iron was nearly in a metalline state. In that state, so extremely divided, and applied dissolved in water at such a temperature, there does not arise a doubt that most of the medicinal effects of these waters may be with confidence attributed to that cause."

What, however, let us ask, is an *almost* volatile state of iron? Is not the ingenious author aware that he is throwing out an idea which sound chemistry will not support? Is this supposed *volatility* of iron any thing more than that the impregnation with this metal is so slight that it is *soon lost to the senses*; namely, so soon as the heat which it naturally possesses has passed away. That more iron should be detected by a delicate than by a sluggish reagent is not very surprising nor uncommon. Neither do we suppose that the following presumptive argument will be considered as very convincing. "The diseases arising from diversity of climate are no less attended to, than the preservation of the human species by suitable nutriment; nay, the diseases which arise from the use, or abuse of what counteracts the effects of climate, are frequently to be obviated by natural and medicinal means discoverable on the spot. The effect of an effect seems to be provided for; indeed there is no one part of Nature, where such a dispensation cannot be traced. To see, therefore, spring up in the midst of a fertile valley, such a stupendous provision as the mineral waters of Bath, must lead us to an investigation of the most interesting nature, when we consider that they must have been sent to answer some adequate purpose."

The author candidly allows that the practice of the General Hospital does not produce a conviction of the efficacy of these waters in any very decided manner. The reason he admits to be, that other medicines have been combined with the water, so as to throw some uncertainty on the proportion of benefit due to the water itself.

self. This concession is fair, but the sceptical enquirer will ask, why it has been necessary to call in so much auxiliary aid?

We are compelled to remark, that the author has, in more than one instance, allowed his zeal for the reputation of this celebrated spring, to outrun his better judgement. We do not hesitate to say *better* judgement, for many very valuable practical remarks are given in the short compass of these pages, and the discrimination between the lædentia and the juvantia in the different states of the diseases which are treated of, is laid down with much clearness and precision. But the virtue of the Bath waters is a subject of so much importance, that we may be allowed, we trust, to pursue it somewhat further.

"With every one who reflects seriously on the efficacy of the Bath waters in removing the early symptoms of some of the most fatal diseases to which man is subject, their credit will in this respect stand as high as if they removed the complaints when fully formed."

Surely not! An emetic exhibited on the first accession of typhus fever will sometimes, by removing the early symptoms, prevent the further progress of the disease; but ought, therefore, the credit of the remedy to stand as high as if it had removed a fever of a week's standing?

The modus operandi of these valuable waters (for valuable they certainly are) is involved in much obscurity. The author does not attempt to add any thing on this head to the conjectures of other writers. As a chemist, we must acknowledge, we hardly expected that he would have thought the following hypothesis worth a repetition.

"It appears from the observations of M. Mandell, a medical professor at Nancy, that the state in which the iron exists in the Bath waters is the most proper for the cure of chlorosis. I extract the following passage from that respectable monthly publication, the Medical and Physical Journal. M. Mandel explains the recent system of Dr. Rollo, who maintains that the greater or less quantity of oxygen in the blood, is the cause of several disorders. Without overturning this principle, he opposes the author's application of it to certain diseases, particularly chlorosis, which, he says, is determined by the smallest quantity of oxygen, and against which he recommends the metallic oxyds, as the most proper to furnish the oxygen. He, on the contrary, proves that this disease is subdued by medicines calculated to lessen the quantity of oxygen, rather than increase it; that it is cured by iron which has undergone no preparation, but the minutest division of its particles; whence he concludes that we ought not to consider oxygenation, but rather ferrugination, as the efficient cause of cure."

Granting the efficacy of abstracting oxygen from the blood in chlorosis, and the de-oxygenating power of the iron in the Bath water, and making the most ample allowance for the quantity of iron ever asserted to be contained in this water; let the ingenious author, for a moment, calculate the proportion between the alleged cause and effect, and he must see that there is a degree of extravagance in hypothesis which excludes all other sentiment but that of ridicule.

---

*The Report on the Cow-Pock Inoculation from the Practice at the Vaccine-Pock Institution, during the Years* 1800, 1801, *and* 1802, *read at the General Meeting of the Governors, February* 7, 1803, *at the Shakspeare Tavern, written by the Physicians to the Institution*; *to which are prefixed, two painted Engravings of Cow-Pock and other Eruptions.* London, pp. 136. 8vo.

It is not often that a public charity can present to its governors and to the public at large, so interesting and every way valuable a report as the present. Much is owing undoubtedly to the high importance and the comparative novelty of the subject, and a great degree of praise is also due to the Medical Directors of the Institution for the earnest attention and philosophical spirit of inquiry which are exhibited in these pages.

This institution, the plan of which was first agitated at Dr. Pearson's house in December, 1799, has now subsisted for three entire years, during which time 1202 patients have been inoculated, namely 317 on the first year, 287 on the second, and 569 on the third. On this sum total the reporters make the following observations. "We are aware that a greater number of persons will be expected to have been inoculated for the cow-pock than are above stated; but it must be considered, that the house for this practice is not situated in a part abounding with poor people; and when it is also considered, that the grand objects of the institution were the preservation of the succession of matter, for the public at large, and for determining the laws of action of the vaccine poison, by accurate registers of observations, it is hoped it will be found, that these points have been completely, or at least in great part attained. With respect to the former of these last-mentioned objects, we have the satisfaction to report, not a week, and very often not a day has passed, without applications being made to the institution for matter to supply great part of the metropolis, and still more extensively, provincial practitioners. In particular, here has been the appointed office for supplying the navy and army by order of government. Nor have the benefits of the institution in this respect been confined to our own country, for France, Germany, Russia, Portugal, Italy, different parts of Asia, Africa, and America, and the West-Indies, have all been furnished with matter from this source. In no instance that has come to our knowledge has our matter been followed by any other effect than the cow-pock; but in a few cases, the small pox, from the intervening agency of that poison (although at first confounded with the cow-pock) has taken place. The chicken-pox, measles, and ulcerous sore throat, which sometimes sometimes occurred during the cow-pock, were too obvious to be imputed to the vaccine poison."

To those only who imagine that the vast heterogeneous mass of population which constitutes *the public*, will instantly act with the vigour and unanimity of a select set of enlightened individuals, can it be a matter of surprize that the bulk of the inhabitants of this kingdom should have been, and should still remain, stupidly indifferent to the benefits of vaccine inoculation, after all reasonable evidence had been laid before them. If however the present report should present a scanty sum total of operations, it exhibits a rich collection of facts selected from the means of observation that have been afforded, which, for the purpose of observation at least, have been amply sufficient. The reporters have thrown together these remarks in the form of propositions or aphorisms, which are here given along with their explanatory comments and annotations.

It would only fatigue our readers again to lay before them the *principia* of vaccine inoculation, with most of which they must long have been familiar. We shall therefore only select a few passages that offer any novelty or refer to points still controverted. "The red areola during the beginning and early part of the scabbing process generally took place, but when it was absent in other respects the pock was the usual one; and the susceptibility of the small pox *was equally destroyed as when the most extensive erythema appeared on the inoculated part.*" A little further on this opinion is explained somewhat more at large. "The areola is neither essential in the same, nor different constitutions; nor connected with the fever; nor with the age of the matter; nor with the quantity of it; nor with the mode of inoculation; but with apparently the state of the skin, in even different arms of the same person; for in the same person, one arm had borne a pock with a large red areola, and the other had a pock with none at all; some have had no areola, and yet a fever; and others no observable fever, yet there was a considerable areola.

"The cicatrix is not always a mark of security, though the absence of it is a certain test that the preservative power of vaccine inoculation has not been secured."

When any symptoms of general fever occur during inoculation, if they happen on the sixth, seventh, and eighth days, the reporters ascribe them to teething or some accidental cause; if later, to the true vaccine disease.

"A disease from various morbific poisons may occur at any period between the time of vaccine inoculation, and the time of the vaccine constitutional affection." The coincident infection of small pox, which has not unfrequently occurred, and which was so satisfactorily explained by Dr. Woodville, is here particularly alluded to. The authors would also infer from this, that the supposed axiom, that two constitutional diseases cannot exist at the same time, is erroneous, and has led to a false supposition of an eruptive species of cow-pox. And yet we may remark, that the very circumstances cumstances of the modification in the appearance of the vaccine pustule produced by the operation of small-pox infection, might be urged in *support* of the disputed axiom. The succeeding proposition, indeed, is more to the point; it is, that "any disorder whatever may take place at any period of the vaccina, except, after a certain progress, the variola."

The reporters here instance a variety of disorders of children that have occurred during the vaccine infection. This is a most important subject of collateral observation, to which all practitioners ought to attend with care. We believe it still open to future investigation.

"The animal economy, for the most part, does not seem to be either bettered or injured by going through the vaccina"

The only consequences of this inoculation hitherto observed, have been, according to the reporters, suppurative swelling of the axillary glands, in very rare instances; and in children not previously subject to eruptions, occasional eruptive vesicles of different forms, for a year or more afterwards.

"Persons who have already gone through the Vaccina, are unsusceptible of it a second time.

"No difference could be perceived in the agency of the vaccine matter according to the age of the pock, or to the presence and absence of areola, except in certain early stages of it, being more efficacious than others." As this relates to a point of practice of considerable importance, and as the opinions of many inoculators will be at issue upon it, we shall give the remarks of the reporters on this subject, and our readers must judge for themselves.

"It was the usage of the Institution almost always to inoculate with matter of the 8th, or 11th and 12th days; and from our ample experience we affirm, that if the pock of the 11th or 12th day was not yet in the scabbing stage, or was but just beginning, the matter was equally efficacious with that of the 8th day; yet, if the pock had begun to scab, or was advanced to that state, this old matter oftener failed than younger matter: but when the matter of such old pocks failed to excite the vaccina, considerable inflammation, or phlegmonous eruption or pimple (which is improperly called spurious cow-pock) did not more frequently occur than from younger matter of the distinct cow-pock, when it failed; and the bad consequences did not ensue more frequently from such old matter, than from matter of the 8th day, or earlier.

"Whether the matter was taken from a pock which had an areola or not, the effect was not on that account different. We have used matter, when it could be had, as early as the 5th day, but it excited the vaccina with the usual appearances; and certainly it was not, on account of the age of the matter, more mild."

"Matter from a pock still containing lymph, though as late as the fifteenth day; and even pus when the *vesicle* had become a *putsule*, generally produced no effect at all except like that of a common scratch; but sometimes it took effect, and then it produced the genuine distinct vaccina.

"In

"In the same arms, matter of the 8th day and of the 12th has been employed, and the event was, that each sort excited the genuine and similar vaccine pocks; and the same effects were seen from matter, so inoculated, of the 11th and 15th days."

Many other important points and remarks are contained in this volume, which is altogether one of the most valuable documents on vaccine inoculation that has appeared, since the publication of those whereby the practice was first established. Two beautiful plates are prefixed, exhibiting vaccine and variolous pustules, and some varieties and anomalies that are described in the course of the book.

We do not consider it to fall within the office of this Retrospect to make any observations on the Plan of the Institution; and we also most willingly avail ourselves of this plea, to pass over, without remark, a circumstance alleged against the conduct of the Discoverer of Vaccination, which the Reporters have thought it necessary here to introduce.

---

